# Effects of Sorafenib and Quercetin Alone or in Combination in Treating Hepatocellular Carcinoma: In Vitro and In Vivo Approaches

**DOI:** 10.3390/molecules27228082

**Published:** 2022-11-21

**Authors:** Suzan Abdu, Nouf Juaid, Amr Amin, Mohamed Moulay, Nabil Miled

**Affiliations:** 1 Department of Biological Sciences, University of Jeddah, Jeddah 23445, Saudi Arabia; sbabdou8@uj.edu.sa; 2 Biology Department, UAE University, Al Ain 15551, United Arab Emirates; a.amin@uaeu.ac.ae; 3 Embryonic Stem Cell Research Unit, King Fahd Medical Research Center, King Abdul Aziz University, Jeddah 22252, Saudi Arabia; moulaay.l@gmail.com; 4 Functional Genomics and Plant Physiology Research Unit, Higher Institute of Biotechnology Sfax, University of Sfax, BP261 Road Soukra Km4, Sfax 3038, Tunisia

**Keywords:** HCC, sorafenib, quercetin, inflammation, necrosis

## Abstract

Sorafenib is the first drug approved to treat advanced hepatocellular carcinoma (HCC) and continues as the gold-standard therapy against HCC. However, acquired drug resistance represents a main concern about sorafenib therapy. The flavanol quercetin found in plants has shown great anti-cancer and anti-inflammatory properties. In this work, quercetin was used as a therapeutic agent alone or in combination with a sorafenib chemotherapy drug to improve the routine HCC treatment with sorafenib. The in vitro and in vivo results presented here confirm that quercetin alone or in combination with sorafenib significantly inhibited HCC growth, induced cell cycle arrest and induced apoptosis and necrosis. Further molecular data shown in this report demonstrate that quercetin alone or combined with sorafenib downregulated key inflammatory, proliferative and angiogenesis-related genes (*TNF-α, VEGF, P53* and *NF-κB*). Combined quercetin/sorafenib treatment markedly improved the morphology of the induced liver damage and showed significant antioxidant and anti-tumor effects. The advantage of combined treatment efficacy reported here can be attributed to quercetin’s prominent effects in modulating cell cycle arrest, apoptosis, oxidative stress and inflammation.

## 1. Introduction

The most frequent primary liver cancer is hepatocellular carcinoma (HCC), which is also the third leading cancer killer globally [1]. Risk factors for it include exposure to aflatoxin, excessive alcohol use, chronic hepatitis B and C virus infection, metabolic syndrome, obesity, diabetes and non-alcoholic fatty liver disease. These causes, either singly or in combination, damage the liver over time, resulting in genetic and epigenetic changes that alter several microenvironmental parameters [2].

Tyrosine kinase inhibitors are the standard medications for individuals who cannot benefit from transarterial chemoembolization (TACE), interventional curative therapy such as radiofrequency or microwave ablation, or surgical resection [3,4]. Sorafenib is a tyrosine kinase inhibitor authorized by the US Food and Drug Administration in 2007 to treat individuals with advanced HCC [5,6]. Vascular endothelial growth factor receptors (VEGFRs) and platelet-derived growth factor receptors (PDGFRs) in endothelial cells and pericytes can be targeted to reduce angiogenesis and cell proliferation via blockage of tumor cells’ B-RAF and RAF1 of the mitogen-activated protein kinase (MAPK) pathway [7,8].

As a result, compared to supportive treatment, sorafenib can extend overall survival by roughly three months [9]. However, a larger fraction of the sorafenib dosage for oral administration in pharmaceutical form travels via either oxidation or glucuronidation, limiting its effectiveness and potency in the treatment of HCC patients [10]. Additionally, clinical trials have demonstrated that roughly 50% of an oral sorafenib dosage is excreted unchanged [11]. Some individuals’ low sorafenib serum levels raise the possibility that oral treatment may not be sufficient to produce reliable and effective therapeutic effects [12].

Antioxidants have been used extensively for the prevention of diseases [13,14], as anti-cancer agents [15] and to reduce drug resistance in HCC experimental models [16]. Quercetin is a flavonoid found in plants, fruits and vegetables that has positive benefits on a number of human illnesses [17].

Quercetin has demonstrated antioxidant, anti-inflammatory and anti-cancer activities. Its impact on both in vitro and in vivo HCC models has been investigated. Quercetin has the ability to control mechanisms that contribute to the development of HCC, including inflammation, migration, apoptosis, fibrosis, angiogenesis [18] and in the involved signaling pathways as well [19]. Through the reduction in reactive oxygen species (ROS) and a rise in the activity of the antioxidant system, quercetin controls oxidative stress, a condition that is directly linked to the development of cancer [20].

Additionally, quercetin modifies the activity of nuclear factor kappa B (NFκB) and suppresses inflammatory enzymes such as cyclooxygenases (COX) and lipoxygenases [21]. Its anti-proliferative activities have been linked to cell cycle arrest induction, the inhibition of cyclin D1 expression, the induction of CDK inhibitors such as p21 and p27, as well as the induction of apoptosis [19,22,23]. Its anti-cancer effects have also been strongly linked to metabolic activity inhibition, cell death induction through the activation of caspases and the blockage of survival signals controlled by either PI3K/AKT/ERK or JACK2/STAT3 signaling pathways [24,25].

Additionally, a growing body of research demonstrating quercetin’s antitumor properties places it as a prospective anti-cancer agent, not only as a stand-alone therapy but also as a means of enhancing currently available therapeutic choices for advanced HCC. In this study, we assess quercetin’s potential as an anti-carcinogenic agent in the cell line and in a rat model.

## 2. Materials and Methods

### 2.1. Ethical Declaration

All tests were conducted in compliance with the ethical principles of animal testing, as well as the technical criteria for the care, production and utilization of laboratory animals of King Fahd medical research center under the authorized protocol (number # 677-20).

### 2.2. Chemicals

The DEN (Cat No: N0258), 2-AAF (Cat No: A7015), quercetin (Cat No: Q4951) and sorafenib (from pharmacy NEXAVAR 200MG for animals and for cell line Cat No: HY-10201) were purchased from SIGMA; (Sigma Chemical Co., Saint Louis, MO, USA). All chemicals utilized were of a high investigative degree, with 98% purity.

### 2.3. Animals

A total of 40 Wistar albino-strain adult male rats weighing 160–200 g and aged 10–12 weeks were procured from the animal house of the King Fahd Medical Research Center (KFMRC) in Jeddah, Saudi Arabia. Throughout the experiment, animals were kept in polypropylene cages (57 × 35 × 20 cm) in a room with a controlled temperature of 25 °C, regular illumination of a 12 h light/dark cycle and relative humidity ranging from 60% to 70%. Throughout the trial, all animals received ad libitum feeding access to regular food and water and their health condition was monitored daily. After one week of acclimation prior to the commencement of the experiment, the rats were separated into two groups (7 normal and 33 HCC induced rats).

### 2.4. Experimental Protocol

The chemically induced HCC rat model was made as described by Abdu et al. [26]. HCC was begun by a single intraperitoneal (IP) injection of diethylnitrosamine (DEN) dissolved in normal saline at a dosage of 200 mg/kg body weight [27]. All rats were treated to a 3-day fasting followed by a one-day re-feeding after start. One week following DEN therapy, rats were given six intra-gastric doses of 2-acetylaminofluorene (2-AAF) (30 mg/kg in 1% Tween 80) for one day, then one dosage per week for four weeks to promote hepatocarcinogenesis. After the successful induction, two rats had died, and three rats were sacrificed to detect the development of cancer through physiological and histological analysis, the remaining rats (28) were randomly divided into four subgroups.

Group I. Contains (7) rats used as controls that were given distilled water (5 mL/kg) and were given a single IP saline injection (in the induction period).

Group II. Contains (7) rats that served as (HCC) while feeding on regular meals and water.

Group III. Contains (7) HCC-induced rats subjected to intra-gastric administration of quercetin (50 mg/kg /day in 1% Tween 80), according to Li et al. [28].

Group IV. Contains (7) HCC-induced rats that were administered with sorafenib (7.5 mg/kg /day in 1% Tween 80), according to Bashmail et al. [29].

**Figure 1:**
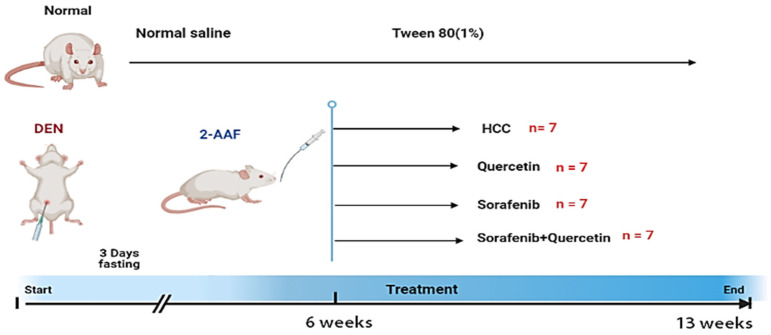
Induction of HCC and quercetin/sorafenib treatment. Diethylnitrosamine (DEN) was used as the beginning agent of carcinogenesis. Following that, 2-animofluorene (2-AAF) was given as a promoting agent. All animals were euthanized after six weeks of post-carcinogenic treatment.

Group V. Contains (7) HCC-induced rats that were administered with sorafenib and quercetin. The same dosages were used in a combination therapy, in which sorafenib was given first, followed by quercetin two hours later. (Figure 1).

Upon six weeks of HCC induction and six other weeks of treatment, all rats were anesthetized. Animals were euthanized after blood samples were taken using retro orbital puncture.

The rats’ body weights were determined before actual commencement of the experiment and recorded after each week along the experimental period of 13 weeks. Ratios of body weight and liver indexes were calculated as follows:

$$\mathrm{Ratio}\;\mathrm{of}\;\mathrm{total}\;\mathrm{body}\;\mathrm{weight}\;\mathrm{change}\;\left(\%\right)=\frac{\mathrm{Final}\;\mathrm{weight}\;–\;\mathrm{Original}\;\mathrm{weight}}{\mathrm{Original}\;\mathrm{weight}\;}\times 100\;\mathrm{liver}\;\mathrm{index}\;(\%)=\frac{\;\mathrm{Liver}\;\mathrm{weight}}{\mathrm{Final}\;\mathrm{body}\;\mathrm{weight}\;}\times \;100$$

### 2.5. Serum Biochemical Markers of Liver Damage Measurement

Serum levels of conjugated bilirubin, total cholesterol (TC), total protein (TP), alanine aminotransferase (ALT), aspartate aminotransferase (AST), triglyceride (TG) and alkaline phosphatase (ALP) were all measured using the rat ELISA kits from BioSource USA and performed in accordance with the instructions and steps outlined in the internal kit’s bulletin.

### 2.6. Determination of Inflammation Biomarkers

Rat ELISA kits were used to evaluate the serum levels of inflammatory markers interleukin 6 (IL-6) and c-reactive protein (CRP).

### 2.7. Assessment of Liver Oxidative Stress

Liver tissues were homogenized for 90 s in buffer (1:10, *w*/*v*) containing 100 mM KCl, 100 mM potassium buffer (pH 7.4), 1 mM EDTA and then homogenates were centrifuged at 10,000× *g* for 30 min at 4 °C to obtain supernatants. Using commercial kits, the supernatants were utilized to determine the concentrations of glutathione (GSH) and lipid peroxidation biomarker (MDA). The protein content in the supernatant was determined using the Lowry et al. technique [30].

### 2.8. Determination of Tumor Marker Levels

ELISA Kits was used to assess serum levels of protein induced by vitamin K absence-II (PIVKA-II) and the liver cancer biomarker fetoprotein (AFP).

### 2.9. Histological and Immunohistochemical Analysis

To make tissue blocks, liver tissue samples fixed in 10% neutral-buffered formalin solution were dried in ethanol, cleaned in xylene, then embedded in paraffin. The latter were sectioned (4–5 m) and the slides stained with hematoxylin and eosin (H&E) or immune-stained. Ki67 immunohistochemical staining was conducted on 4 m paraffin sections mounted on positively charged slides using a Benchmark GX machine with Standard Cell Conditioning (CC1) and baked for 1 h in a 60 °C oven. The sections were treated for 16 min at 37 degrees Celsius with the pre-diluted primary antibody, rabbit anti-Ki-67 (clone 30-9). The antigen–antibody response was identified with 3,3-diaminobenzidine (DAB) using the Ultra View Universal Dab Detection Kit. The sections were counterstained with hematoxylin for 4 min before being dehydrated in a graded series of ethanol, cleaned with xylene, and the slide covered with DPX.

### 2.10. Gene Expression Analysis

An RNA extraction kit was used to isolate total RNA from tissue samples kept at −80 °C (Cat No.: R1200 Solar bio, Beijing, China). The cDNA was created by reverse transcription of RNA using Quantiscript reverse transcriptase, as directed by the manufacturer (Cat No.: RP1100, Solar bio, Beijing, China). Based on the references listed in Table 1, specific primers for tumor necrosis factor alpha (TNF-a), vascular endothelial growth factor (VEGF), tumor suppressor (p53) and nuclear factor kappa light-chain enhancer of activated B cell (NFB) genes were chosen. Quanti Tect SYBR Green qPCR Master Mix was used in a StepOnePlus Real-Time PCR system (Applied Biosystems, USA) and reaction cycles (Reaction mixtures were 191 incubated for 10 min at 95 °C, followed by 40 cycles of 15 s at 95 °C, 1 min at 60 °C and, finally, 15 s at 95 °C, 1 min at 60 °C and 15 s at 95 °C). The critical threshold (Ct) of target genes was standardized against the critical threshold (Ct) of the internal control (GPDH). The concentrations were represented as a percentage of the normal control samples. Table 1 shows the primers used for real time qPCR.

### 2.11. Cell Growth

The American Type Culture Collection (ATCC) provided the Hepatocellular Carcinoma cell line (HepG2) (Manassas, VA, USA). The cell line was grown in DMEM media supplemented with 10% fetal bovine serum (Sigma Aldrich), containing 1% of 100 U/mL penicillin and 100 g/mL streptomycin (Sigma Aldrich), and incubated at 37 °C in a humidified 5% CO_2_ environment. Cells were subcultured every 3 days with 0.25% trypsin.

### 2.12. Cytotoxicity Assay

The MTT (3-(4, 5-dimethyl thiazol-2yl)-2, 5-diphenyl tetrazolium bromide) test was used to investigate the cytotoxicity of quercetin and sorafenib on HepG2 cells [36]. cells (10,000/well) were plated in 96-well plates with 180 L of full-growth media. For 48 h, the attached cells were treated with various doses of quercetin (20 μM, 60 μM, 100 μM, 140 μM, 180 μM and 220 μM) and Sorafenib (5 μM, 10 μM, 15 μM, 20 μM, 30 μM and 40 μM) or a sorafenib/quercetin equimolar combination (6.25 μM, 12.5 μM, 25 μM, 50 μM and 100 μM). After incubation, the media were carefully withdrawn from each well and rinsed with 90 L of fresh culture medium before adding 10 L of 3-[4,5-dimethylthiazol-2-yl]-2,5-diphenyltratrazolium bromide (MTT) solution (Sigma Aldrich) and continuing the culture for 4 h in a 5% CO_2_ incubator. Following that, 1 mL of DMSO (solubilizing reagent) was added to each well, stirred and incubated for 45 s. The existence of live cells was shown by the creation of formazan crystals, which produced a purple tint. The suspension was transferred to the cuvette of a spectrophotometer, and the OD (optical density) readings were read at 570 nm using DMSO as a blank. The concentration necessary for a 50% inhibition of viability (IC50) was visually estimated by graphing the concentration of the medication on the *X* axis and relative cell viability on the *Y* axis.

$$\mathrm{Cell}\;\mathrm{viability}\;\left(\%\right)=\frac{\mathrm{Mean}\;\mathrm{OD}}{\mathrm{Control}\;\mathrm{OD}}\times \;100$$

The combination index (CI) was determined using the following method for combined therapy with sorafenib and quercetin:

$$CI=\frac{IC50\;of\;sorafenib\;combination}{IC50\;of\;sorafenib\;alone}+\frac{IC50\;of\;Quercetin\;combination}{\begin{array}{c} IC50\;of\;Quercetin\;alone \end{array}}$$

The type of drug interaction is described as synergism if *CI* < 0.8, antagonism if *CI* is greater than 1.2 and additive if *CI* is between 0.8 and 1.2 [37].

### 2.13. Cell Cycle Examination

Flow cytometry was used to investigate the influence of quercetin and sorafenib on the cell cycle distribution of HepG2 cells. For 48 h, the cells were treated with either free media (control) or the pre-determined IC50 of quercetin, sorafenib, or a quercetin/sorafenib combination. After incubation, the cells were trypsinized and washed twice with ice-cold PBS before being re-suspended in 0.5 mL of PBS. Two milliliters of 60% ice-cold ethanol were added, and the cells were fixated at 4 °C for 1 h. After being washed, the fixed cells were resuspended in 1 mL of PBS containing 50 g/mL RNase A and 10 g/mL propidium iodide. The cells were examined for DNA content after 20 min of incubation in darkness at 37 °C using an FL2 (ex/em 535/617 nm) signal detector (ACEA NovocyteTM flow cytometer) (ACEA Biosci-ences Inc., San Diego, CA, USA). There were around 12,000 occurrences per sample. ACEA Novo-ExpressTM software was used to calculate cell cycle distribution (ACEA Biosciences Inc., San Diego, CA, USA).

### 2.14. Apoptosis Assays

The effect of euercetin, sorafenib, and quercetin/sorafenib on apoptosis and necrosis in the examined cell lines was assessed using flow cytometry and the Annexin/V-FITC apoptosis detection kit (Annexin V-FITC/PI Apoptosis Detection Kit, CA1020, Solarbio, China). In brief, the cell line was treated for 48 h with the corresponding IC50 of quercetin, sorafenib, or both quercetin and sorafenib. Following that, the cells were trypsinized, washed twice with ice-cold PBS and re-suspended in 0.5 mL of annexin/V-FITC/PI solution for 30 min in the dark, per the manufacturer’s procedure. The cells were stained at room temperature before being injected into the ACEA Novo-cyteTM FCA (ACEA Biosciences Inc., San Diego, CA, USA) and examined for FITC and propidium iodide fluorescent signals using FL1 and FL2 detectors (ex/em 488/530 nm for FITC and ex/em 535/617 nm for PI). Approximately 12,000 events were collected, and positive FITC and/or PI cells were counted and computed using ACEA NovoExpressTM software (ACEA Biosciences Inc., San Diego, CA, USA). Each treatment was done three times, and the data reflect the means and standard deviations of three replicates.

### 2.15. Statistical Analysis

The study’s data were analyzed using IBM SPSS Statistics for Windows, version 23. (IBM SPSS, IBM Corp., Armonk, NY, USA). The Shapiro–Wilk test was used to examine the normal value distribution. The collected value is displayed as the mean +/− standard deviation (SD). One-way analysis of variance (ANOVA) was employed for statistical comparisons, and Newman–Keuls was utilized as a post hoc test, followed by least significant difference (LSD) analysis. The *p* value of 0.05 was deemed statistically significant. ED50 plus V1.0 software was used to calculate the IC50 for all cell lines. All results were shown as the mean standard deviation (SEM) of three replicates (*n* = 3). Prism (V5, Co., San Diego, CA, USA) was used to evaluate the statistical data, and differences between groups were judged significant at * *p* < 0.05.

## 3. Results

### 3.1. Effect of Treatment on Body and Liver Weights

**Figure 2:**
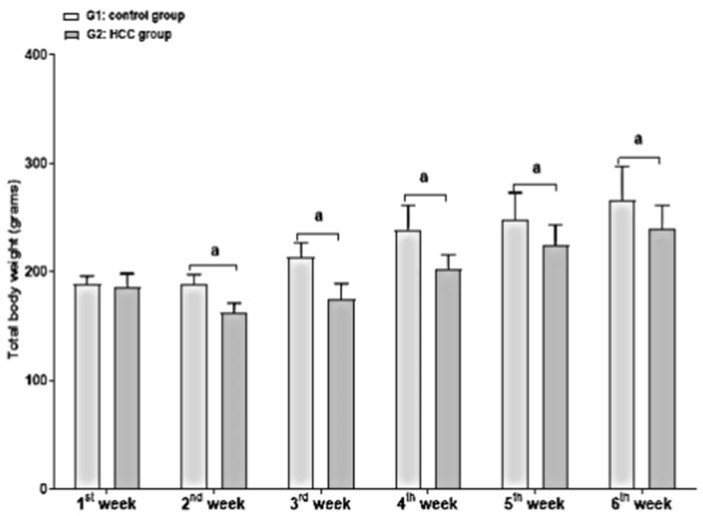
Body weight (grams) during the first to sixth weeks of the control (G1) and HCC-induced rat (G2) groups. a: significance versus G1. Significance at *p* < 0.050.

The rat body weights were measured on a weekly basis along the experimental period of thirteen weeks. Upon HCC induction, the body weight increase of the HCC group (28.16%) was significantly lower (*p* < 0.05) in comparison to the control (40.51%) (Figure 2). When compared to the control, HCC caused a considerable weight reduction. The increase in body weight at the end of week 13 significantly declined in the HCC and quercetin-treated groups in comparison to the control (Table 2) (*p* < 0.05). Following anti-tumor treatment with sorafenib or a sorafenib/quercetin combination, the body weight ratio was restored at week 13. No significant differences were noticed in the liver weight and liver index of the treated and non-treated groups compared to the control animals (*p* < 0.05) (Table 2).

### 3.2. Effect of the Treatments on Liver Function Enzymes and Lipid Profile

**Figure 3:**
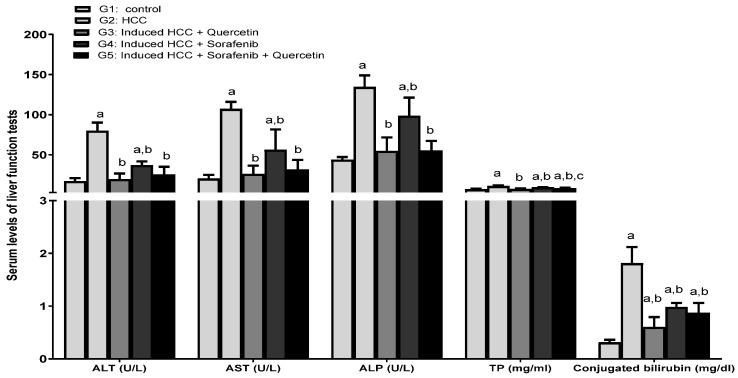
Liver enzyme levels in the serum of different studied groups. Data were expressed as mean +/− standard deviation. Significance was made using one-way ANOVA test followed by least significant test. a: significance versus group 1. b: significance versus group 2; c: significance versus group 4. ALT: Alanine aminotransferase; AST: Aspartate aminotransferase; ALP: alkaline phosphatase; TP: total protein. Significance at *p* < 0.050.

The HCC-induced rats displayed a significant increase in the serum levels of liver enzymes—aminotransferase (ALT), aspartate aminotransferase (AST) and alkaline phosphatase (ALP), total proteins (TP) and conjugated bilirubin (direct bilirubin)—versus the control group (*p* < 0.05) (Figure 3). This is indicative of liver alteration due to carcinogenesis.

Following six weeks of HCC treatment with quercetin, sorafenib or sorafenib/quercetin, the serum levels of enzyme activities were significantly decreased compared to the non-treated HCC group (*p* < 0.05). Treatment with sorafenib partially reduced the activities of these enzymes, whereas using quercetin alone or combined with sorafenib greatly improved the recovery of serum enzymes’ activities to levels close to those of normal animals (G1) (*p* < 0.05).

**Figure 4:**
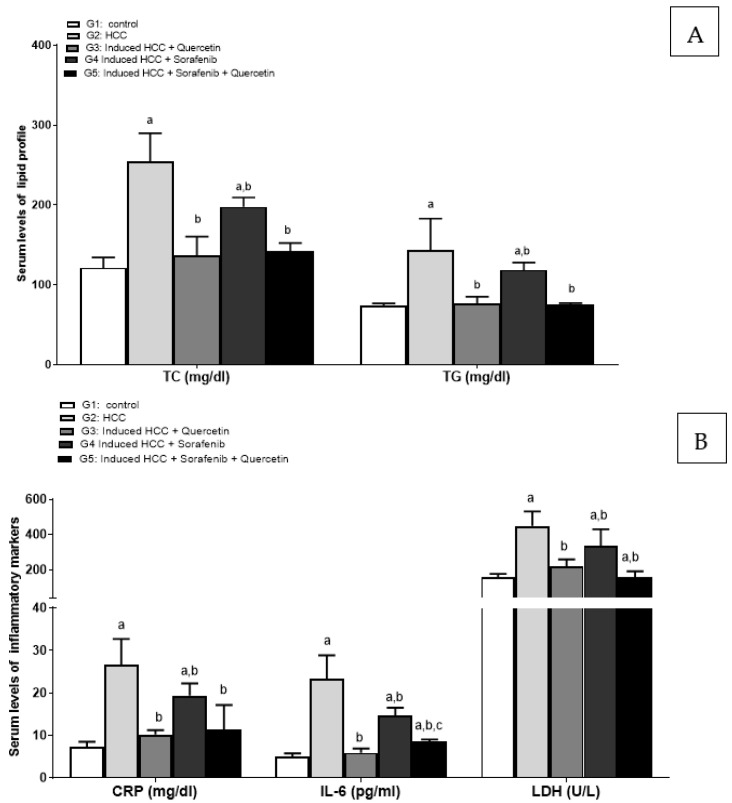
Serum levels of lipid profile (TC and TG) (**A**) and inflammatory markers CRP, IL-6 and LDH (**B**). Data were expressed as mean +/− standard deviation. Significance was made using one-way ANOVA test followed by least significant test. a: significance versus group 1. b: significance versus group 2. c: significance versus group 4. TC: total cholesterol; TG: total triglyceride; CRP: C- reactive protein; IL-6: Interleukin -6; LDH: Lactate dehydrogenase. Significance at *p* < 0.05.

Likewise, HCC induction significantly increased serum lipid concentrations versus the control group (*p* < 0.05). In all treated rat groups, the serum lipid concentrations of triglyceride (TG) and total cholesterol were significantly decreased versus the HCC group (G2) (*p* < 0.05). While serum lipid rates decreased slightly upon sorafenib treatment, they decreased markedly for rats treated with quercetin alone or the sorafenib/quercetin combination, compared to the HCC group (*p* < 0.05) (Figure 4A).

### 3.3. Effect on Inflammatory Markers

The untreated HCC rats showed a significant increase in serum levels of inflammatory markers: C-reactive protein (CRP), interleukin 6 (IL-6) and lactate dehydrogenase (LDH) compared to the non-treated rat group (*p* < 0.05). Meanwhile, all treated groups (G3, G4 and G5) displayed significantly decreased levels of serum inflammatory markers versus the HCC group (G2) (*p* < 0.05). The administration of any of the anti-cancer drugs significantly decreased the levels of inflammatory markers compared to the non-treated HCC group (*p* < 0.05). Although all drugs tended to reduce the inflammatory markers to various extents, the sorafenib/quercetin treatment was the most effective in lowering the serum levels of CRP, IL-6 and LDH (Figure 4B).

### 3.4. Effect on the Levels of Oxidative Stress

**Figure 5:**
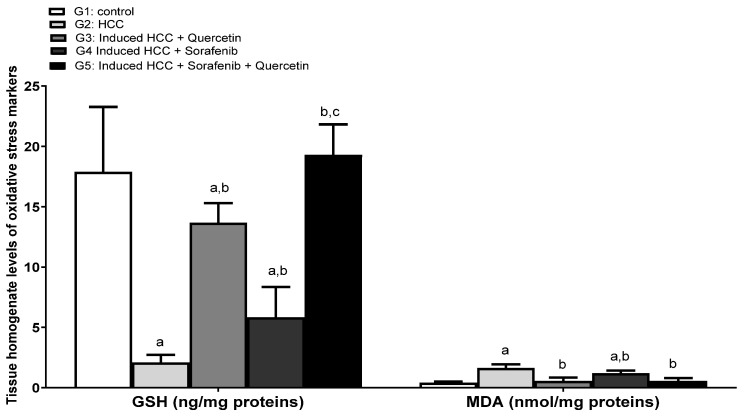
Tissue homogenate levels of oxidative stress markers in different studied groups. Data were expressed as mean +/− standard deviation. Significance was made using one-way ANOVA test followed by least significant test. a: significance versus group 1. b: significance versus group 2. c: significance versus group 4. GSH: glutathione; MDA: malondialdehyde. Significance at *p* < 0.050.

Glutathione (GSH) is the major redox couple in animal cells. Glutathione deficiency contributes to the oxidative stress involved in aging and the pathogenesis of many diseases, such as liver diseases, cancer and diabetes [38]. Moreover, the detection of malondialdehyde (MDA) was traditionally used as an indicator of lipid peroxidation. The levels of glutathione (GSH) decreased significantly in the HCC group and was accompanied by an increase in the content of MDA, compared to the control group (*p* < 0.05) (Figure 5). This is explained by oxidative stress caused by cancer development.

Treatment with sorafenib alone failed to adjust dramatic changes in oxidative stress markers in the HCC group. A drastic improvement of these indicators was observed in the livers of rats treated with quercetin alone compared with the results of the HCC group. Interestingly, the combined sorafenib/quercetin treatment succeeded to restore the GSH rates to normal levels.

### 3.5. Effect on the Levels of Tumor Prognostic Markers

Protein Induced by Vitamin K Absence (PIVKA-II) is an abnormal prothrombin precursor produced by hepatocellular carcinoma cells, used as a biomarker for HCC surveillance. PIVKA-II and alpha-fetoprotein (AFP) are produced independently in HCC cells and serve as complementary markers for HCC. Their combined use demonstrated increased specificity and sensitivity than using either marker alone [39]. The serum levels of PIVKA-II and liver AFP were significantly increased upon HCC induction (*p* < 0.05).

**Figure 6:**
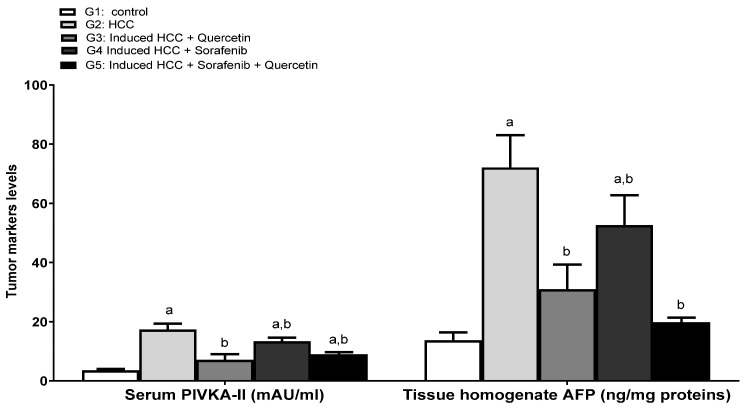
Tumor marker PIVKA-II and tissue homogenate of AFP in different studied groups. Data were expressed as mean +/− standard deviation. Significance was made using one-way ANOVA test followed by least significant test. a: significance versus group 1. b: significance versus group 2. PIVKA-II: Protein Induced by Vitamin K Absence; AFP: alpha-fetoprotein. Significance at *p* < 0.050.

The various treatments resulted in a significant decrease in the levels of tumor markers compared to the HCC group (*p* < 0.05). Sorafenib slightly decreased the levels of tumor biomarkers (*p* < 0.05). Interestingly, quercetin used alone or in combination with sorafenib significantly reduced the levels of tumor biomarkers. Even though both treatments reduced PIVKA-II to comparable levels, the combined treatment was more effective in attenuating AFP rates to values close to those of normal rats (Figure 6). The combination therapy using sorafenib/quercetin was more effective in reducing tumor biomarkers to normal levels (Figure 6).

### 3.6. Effect of the Treatments on Hepatic Tumor Area and Liver Architecture

The photomicrographs of liver sections of the control group showed a normal architecture of the hepatocytes with eosinophilic cytoplasm. The hepatocytes were organized into cell plates separated by sinusoids that radiated from the central vein. An examination of the HCC liver tissue showed histopathological changes marked by increased cell density and coagulative basophilic cytoplasm, increased nuclear-to-cytoplasmic ratios and nuclear atypia. We observed groups of cells and nodules that stand out from the background described, as well differentiated tumors. Architectural abnormality was noticed, with thicker, disorganized hepatocyte cords replacing the normal ones, thin hepatocyte trabeculae or cords (three cells or more in thickness) and solid sheets of cells. The growth patterns were trabecular and solid, and displayed rarely pseudo acinar structures. Clear cell carcinoma, steatosis and inflammation were also observed. Other cytological changes noticed include eosinophilic or hyaline inclusions. Although there were proliferating bile ducts close to the tumors, there were no real portal tracts with the typical triad of the bile duct, hepatic artery and portal vein. Increased sinusoidal congestion and bile production were also noted.

**Figure 7:**
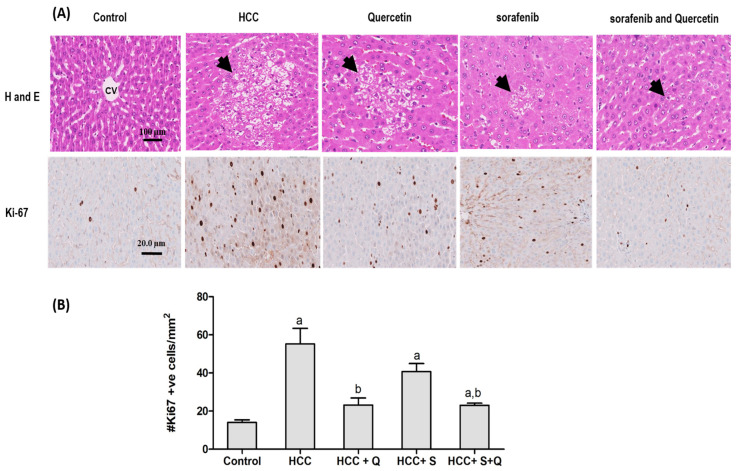
(**A**) Anti-tumor effect of anti-cancer treatments. Representative images of hematoxylin and eosin-stained (H and E) and Ki-67-stained sections of animals’ liver tissues are presented. The control corresponds to normal rats’ liver tissue. HCC is the HCC-induced group. HCC + Q, HCC + S and HCC + S + Q correspond to HCC rats treated with quercetin, sorafenib or a quercetin/sorafenib combination, respectively. Representative areas of tumor nodules and tumor cells are indicated by arrows. (**B**) shows the quantitative analysis of Ki-67 immunoreactive cells in 10 fields of each section of the Ki-67 positive foci and quantitative region analysis of the Ki-67–positive foci ×100 magnification (Scale bar 20× m). The values were evaluated by one-way ANOVA followed by Dunnett’s *t*-test compared to the HCC induced group. Data are represented as mean ± SEM. The letter (a) is used for *p* < 0.05 vs. control group and the letter (b) is used for *p* < 0.05 vs. HCC group.

Treatment with quercetin or sorafenib significantly alleviated liver tissue damage and markedly reduced tumor nodules and tumor cell sheets, inflammation, angiogenesis, cytoplasm basophilic and increased apoptosis, as well as necrosis. Yet, liver tissue corresponding to sorafenib-treated HCC rats showed reduced cell density, with many strands of three cells or more of hepatocytes and pale cytoplasm due to increased lipid vacuoles. The combined sorafenib/quercetin treatment resulted in reduced tumor nodules and cell density with increased necrosis, apoptosis and hyperchromasia. Interestingly, the combined sorafenib/quercetin treatment was mostly powerful in reducing tumor nodules and signs of liver tissue damage, inflammation, angiogenesis and inducing apoptosis and necrosis. This finding strongly suggests that combined sorafenib/quercetin can inhibit the growth of hepatic tumor nodules and restore the liver’s structural integrity (Figure 7).

Nuclear Ki-67 proteins are used to estimate a tumor’s proliferation index, which is a useful tool in diagnosing well-differentiated HCC cells. Dividing cells display a high Ki-67 expression level. The overexpression of this protein observed in the liver section of the HCC group is usually associated with tumorigenesis. The number of Ki-67-expressing hepatocytes of cancer-induced animals increased dramatically compared to the control (Figure 6). Treatment with either sorafenib or quercetin induced a significant decrease in the number of Ki-67-expressing hepatocytes. Nevertheless, the combined treatment was more effective in suppressing Ki67 overexpression (Figure 7).

### 3.7. Effect of Treatments on the Expression of Key HCC Genes

**Figure 8:**
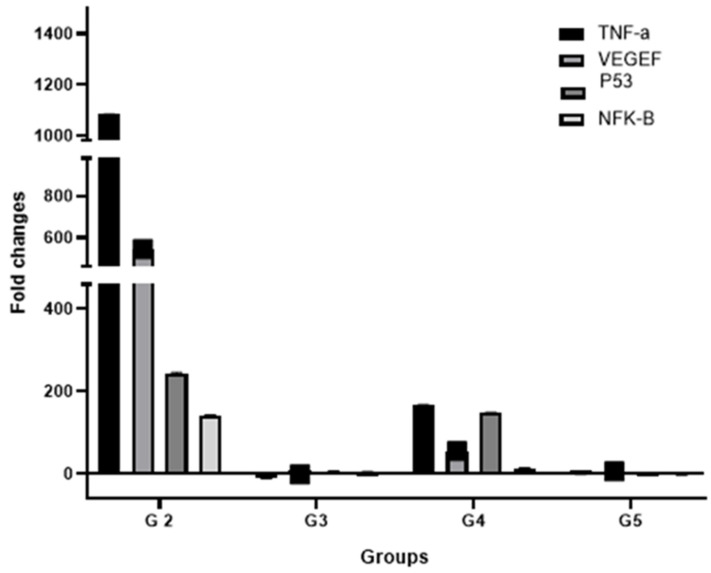
Diagram represents the effect of different drugs on the fold change of gene expression for TNF-a, VEGF, p53 and NFκB as target genes vs GAPDH as reference gene. The fold change corresponds to the relative expression quantification (RQ) if RQ > 1 (upregulation), −1/RQ if RQ < 1 (downregulation) and 0 if RQ = 1 (no regulation).

Tumor necrosis factor-α (*TNF*-*α*), vascular endothelial growth factor *(VEGF*), tumor protein p53 (*TP53*) and *NFκB* genes play a crucial role in cell proliferation and angiogenesis associated with tumor evolution in HCC [17]. *TNF-α* is one of the most important inflammatory cytokines that induces tumor necrosis [40]. It was observed that *TNF-α* expression in HCC is significantly higher than that in normal liver tissue [41]. The expression of p53 and VEGF could reflect the prognosis of liver cancer [42]. Furthermore, the dominant role of *NFκB* in liver cancer development and progression has been demonstrated [43]. *TNFα, VEGF, p53* and *NFκB* were overexpressed upon HCC induction compared to non-induced rats (*p*-value < 0.05) (Figure 8). This is indicative of cancer cell proliferation, angiogenesis and associated inflammation and necrosis (Figure 8). Sorafenib treatment failed to suppress completely gene overexpression (*p* < 0.05), although it was markedly reduced compared to that of HCC-induced rats. Interestingly, treatment with quercetin alone or in combination with sorafenib suppressed the overexpression of *VEGF* and *p53* genes (*p* > 0.05) (Figure 8). Cancer cell processes associated with angiogenesis and proliferation are therefore attenuated. For all treatments, the *TNFα* gene over expression was not suppressed but remained lower compared to the HCC group (*p* < 0.05). This can be explained by the persistence of HCC-induced inflammation despite the treatments. Regarding *NFκB*, quercetin treatment and, to a lesser extent, sorafenib suppressed the gene overexpression (*p* > 0.05). HCC treatment with quercetin alone or in combination with sorafenib downregulated key genes involved in carcinogenesis.

### 3.8. Measurement of the Cytotoxicity of Quercetin and Sorafenib Using the MTT Assay

**Figure 9:**
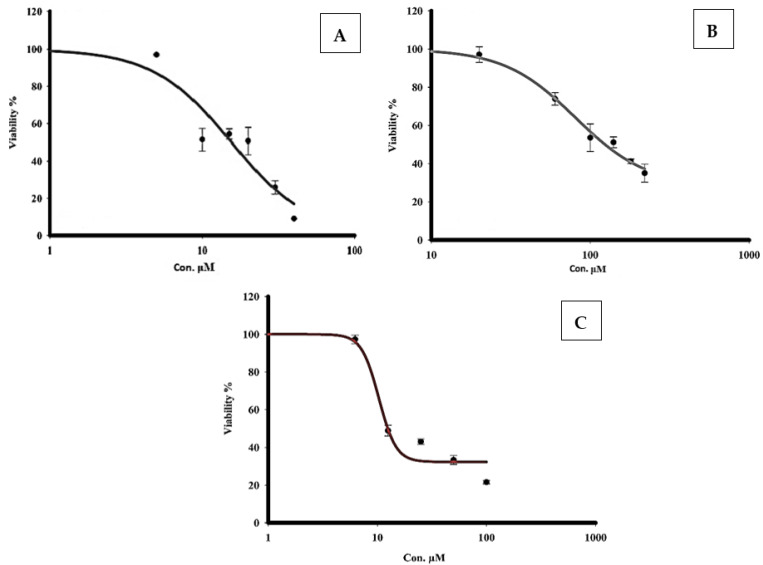
Effect of treatment with sorafenib (**A**), quercetin (**B**) and sorafenib/quercetin (**C**) on HepG2 cell viability. IC50 value corresponds to the concentration that reduces cell viability to 50%. Data are expressed as mean ± SEM for three replicates (*n* = 3).

The cytotoxicity of sorafenib and quercetin against the HCC cell line HepG2 was assessed. Quercetin (20 to 220 μM) or sorafenib (5 to 40 μM) was incubated with HepG2 cells for 48 h. The half maximal inhibitory concentration (IC50) of cell viability was then determined using the MTT assay. The IC50 values corresponding to sorafenib and quercetin were 10.9 μM (Figure 9A) and 107.7 μM (Figure 9B), respectively. To check the combined effect of sorafenib and quercetin on hepatocarcinoma cells, we assayed the effect of various concentrations (from 6.5 μM to 100 μM) of an equimolar mix of quercetin and sorafenib on HepG2 cell viability. The IC50 of the mix sorafenib/quercetin at 48 h was 9.98 μM (Figure 9C). The equimolar combination of sorafenib and quercetin displayed, therefore, a higher inhibitory potential of cell proliferation than the individual drugs. The combination index (CI) is a measure of the interaction between two drugs [29]. CI values lower than 1 are indicative of a synergism. The estimated CI value was 0.54 indicating a synergism between the sorafenib and quercetin.

### 3.9. Cell Cycle

**Figure 10:**
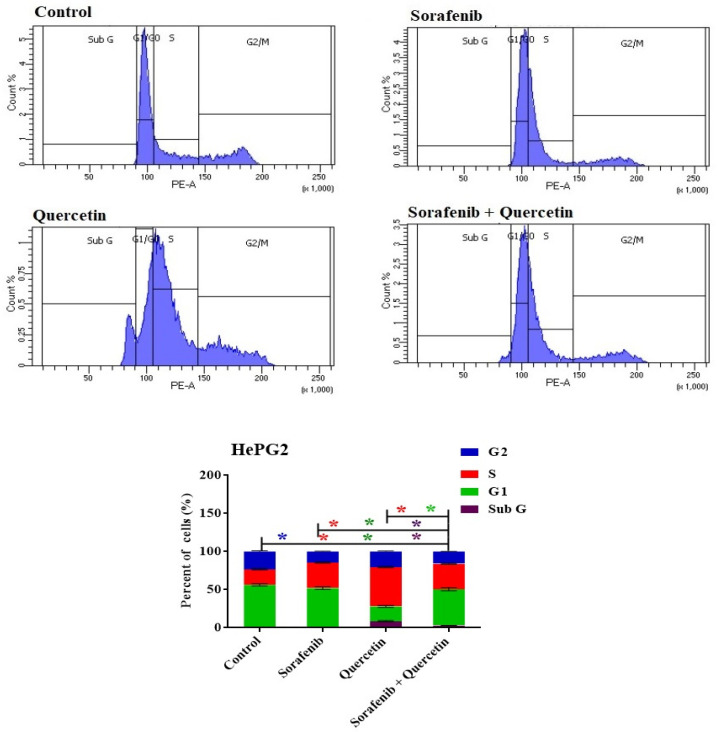
DNA cytometry analysis showing the effects of sorafenib and quercetin on the cell cycle distribution of HePG2 cells. Cells were exposed to sorafenib, quercetin and the mix for 48 h. Cell phases were plotted as percentage of total events. Sub-G cell population was plotted as percent of total events of cells. Data are presented as mean ± SEM for three replicates (*n* = 3). The differences of events from each respective control are considered significant at * *p* < 0.05.

The anti-proliferative capacities of sorafenib and quercetin were assayed against HepG2 cell lines. After incubation for 48 h with each drug or with a combination of both drugs at IC50 values, the cell cycle arrest was checked by analysis of the DNA content using flow cytometry (Figure 10). Arresting the cell cycle at early G1 or G2/M phases results in cell death by apoptosis due to DNA damage and subsequent aberrant mitosis [44,45,46].

The cell cycle steps are monitored mainly at the G1/S boundary, S phase and during the G2/M-phase checkpoints that are under the control of stimulatory signals such as growth factors [47].

While sorafenib arrested the cell cycle at the S phase, quercetin exerted an anti-proliferative effect and arrested the cell cycle of the cancer cell lines in G1 and S phases (Figure 10). The quercetin significantly increased the cell population at the S phase with a reciprocal decrease in the G1 phase (Figure 10). In response to the sorafenib/quercetin combination, the cell population in the S phase significantly increased (Figure 10), indicating an anti-proliferative effect compared to non-treated cells.

Both quercetin and the sorafenib/quercetin combination exerted an anti-proliferative action on HepG2 cells through an apoptosis-inducing property.

### 3.10. Annexin–V-FITC Analysis (Apoptosis Assay)

**Figure 11:**
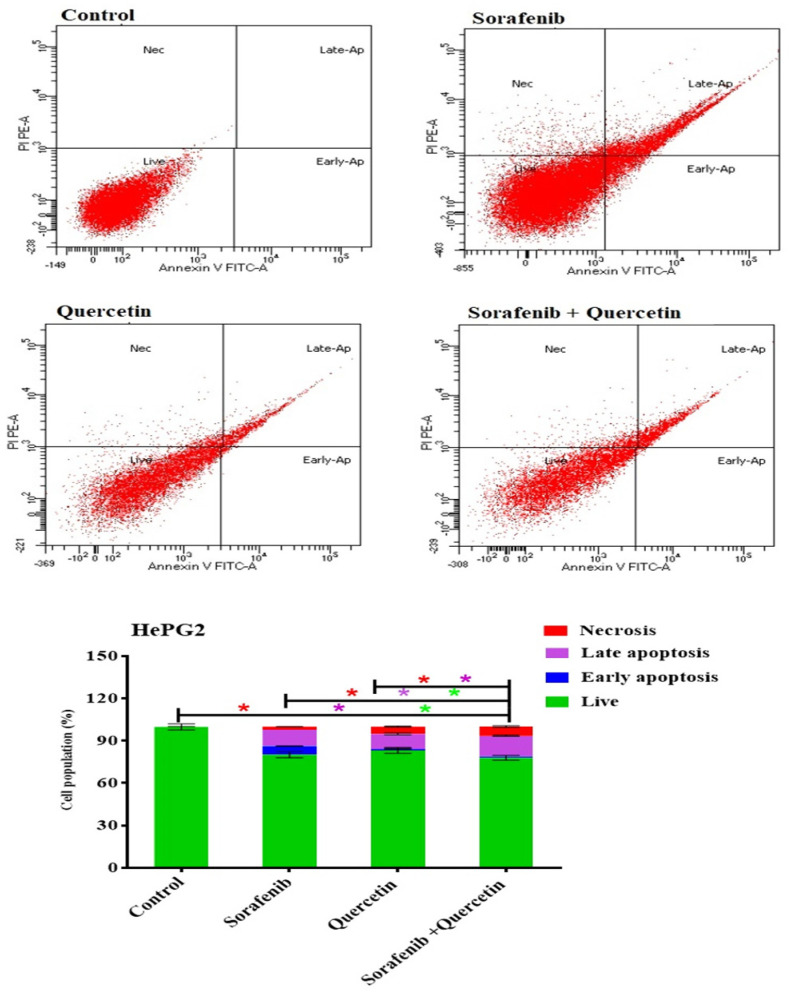
DNA cytometry analysis of Annexin–V-FITC showing representative flow cytometry panels of apoptosis, necrosis and cell vitality of HePG2 in response to 48 h exposure to media only (control) or (sorafenib, quercetin and sorafenib/quercetin, respectively). Cell populations (%), including early apoptosis, late apoptosis, necrosis and total cell death for the different treatments were shown in the lower panel. All data are presented as mean ± SEM for three replicates (*n* = 3). Differences are considered significant at * *p* < 0.05.

Cancer cell death is attributable to necrosis or apoptosis. The HepG2 cancer cell line was incubated for 48 h with sorafenib, quercetin or combined molecules at IC50. The cells were then analyzed using the Annexin–V-FITC, and apoptotic and necrotic cells were differentiated using flow cytometry apoptosis detection assay. Sorafenib, quercetin and the combined treatment significantly increased the number of necrotic and apoptotic cells (Figure 11).

Sorafenib, quercetin and the combined treatment significantly increased the number of necrotic and apoptotic cells. Sorafenib induced cell death by necrosis (2.2%), early apoptosis (6.1%) and late apoptosis (11.6%) (Figure 11). Quercetin increased cell death by necrosis (5.2%) and induced only late apoptosis death (10.6%). The combined quercetin/sorafenib treatment was the most effective in inducing cell necrosis (6.5%) and late apoptosis (14.6%), thus confirming a synergetic effect between both molecules. Unlike sorafenib, quercetin used alone or combined with sorafenib induced low early apoptosis (1% vs. 6.1%). Sorafenib and quercetin induced late apoptosis at close rates (11.6 and 10.6), whereas their combination increased the rate to 14.6%.

## 4. Discussion

Advanced HCC is typically treated with sorafenib to stop angiogenesis, cause apoptosis and decrease cell proliferation. It affects the Mcl-1, angiogenic tyrosine receptors and the MAPK pathway. Sorafenib’s effectiveness has been demonstrated in clinical tests, although there are a number of side effects and a high rate of treatment resistance among patients. The present work confirmed the well-established anti-cancer capability of sorafenib. To enhance its effects, reduce its drawbacks, lower the required dosage, or overcome resistance, sorafenib has frequently been used in conjunction with other therapies in studies. Natural substances such as quercetin have been acknowledged as important agents in the treatment and prevention of cancer. We studied the efficacy of the combination of sorafenib and quercetin to examine the synergistic effect on the development of drugs for cancer chemotherapy. Due to its capacity to control the cell cycle, antioxidant properties, stability of p53 and ability to induce apoptosis, quercetin has the potential to be employed in the treatment of cancer. Quercetin appears to be an important anti-proliferative and anti-cancer drug [48].

In the present study, increased levels of inflammatory markers (CRP, IL-6, LDH) were associated with HCC induction. However, quercetin modulated the inflammation markers. Likewise, in a human hepatocyte-derived cell line, preclinical research has demonstrated that quercetin can drastically lower the levels of inflammation moderators such as NO synthase, COX-2 and CRP [49,50]. Increasing liver proteins and serum lipid rates were associated with liver damage induced by HCC. The combined treatment was more effective than quercetin alone or sorafenib in liver function recovery. Similarly, the combined sorafenib/quercetin treatment was the most effective in reducing oxidative stress, tumor biomarkers, tissue damage and angiogenesis. This treatment was effective in inhibiting the growth of hepatic tumor nodules and restoring the liver’s structural integrity. Sorafenib-treated HCC rats displayed signs of cancer regression with increased lipid vacuoles in hepatocytes. This is in line with relatively high serum lipid contents compared to the other treatments.

Sorafenib could suppress the overexpression of the *NF-κB* gene in rat livers upon HCC induction. Meanwhile, quercetin used alone or combined with sorafenib was able to downregulate *p53*, *VEGF* and *NF-κB* as key genes involved in angiogenesis and cancer development. The *p53* tumor suppressor gene prevents cellular proliferation through cell cycle arrest and apoptosis in response to DNA damage. Increased *p53* levels in HCC rats is concomitant with cancer development. The reduction in *p53* levels in treated animals is likely due to the cell recovery. Our results recall reports that quercetin downregulated the *VEGF* [51] and *NF-κB* [52] genes in cancer cell lines. *VEGF* downregulation reduced angiogenesis and cancer development upon treatment with anti-cancer drugs. Apoptosis induced by quercetin, sorafenib or combined molecules could be explained by various mechanisms, including the downregulation of *NF-κB*, as previously suggested [52], and induction of cell cycle arrest.

Angiogenesis is a key cancer-related process. The present study confirmed that quercetin exerted an anti-angiogenesis effect on HCC, as reported in various cancers [53]. The effect of quercetin as an anti-proliferative agent against HCC through employing a cell cycle arrest mechanism was confirmed, as reported before [54] in ovarian cancer through various mechanisms, including anti-inflammation, pro-oxidation and anti-proliferation. Cell cycle arrest was suggested to be monitored by the inactivation of key enzymes in DNA duplication such as topoisomerase [55]. In the present work, quercetin alone or combined with sorafenib was shown to arrest cell cycle in the S phase and induce apoptosis. Likewise, several anti-cancer agents are known to induce S-phase arrest in various types of human cancer cell lines, including cisplatin [56], mitomycin C [57] and resveratrol [58].

Chemotherapy acts through necrosis rather than apoptosis as a preferred mode of cell death [59]. The present study confirmed a strong potential of quercetin in inducing necrosis in vitro and in vivo. Quercetin is a good option to treat liver cancer alone or combined with another anti-cancer drug, since it does not affect healthy cells while having cytotoxic effects on cancer cells through several methods [60].

By lowering the expression of drug efflux transporters, P-glycoprotein and breast cancer resistance protein, it has been proposed that quercetin enhances the anti-cancer action of sorafenib against breast cancer cells [61]. The combination of sorafenib and quercetin is seemingly a new promising treatment for HCC. We demonstrated in vitro and in vivo that the combination of quercetin with sorafenib synergistically suppressed proliferation, and promoted apoptosis and necrosis. This study displayed a synergy between sorafenib and quercetin in cancer treatment. In the in vivo system, the combination therapy mainly increased the liver recovery after anti-HCC treatment. As sorafenib is the main treatment used, its administration displayed marked necrosis and inflammation as parts of the treatment side effects. The combination therapy was effective in reducing the levels of inflammation and oxidation markers, compared to the treatment with sorafenib alone. The normal liver architecture was also restored after using the combined treatment. Likewise, the combined treatment markedly reduced the liver damage compared to sorafenib. This could be observed in the reduction of liver enzyme levels (Figure 3) and lipid contents in the serum (Figure 3 and Figure 4). In the in vitro system, the combination therapy induced cancer cell apoptosis and cell cycle arrest better than sorafenib (Figure 10 and Figure 11). The combination therapy seems, therefore, more effective than sorafenib in ameliorating the treatment outcomes and limiting the side effects.

## 5. Conclusions

The combination of sorafenib/quercetin produced a synergistic anti-cancer effect both in vitro and in vivo. It decreased HCC cell viability, blocked the progression of the cell cycle, and promoted apoptosis and necrosis. The treatment of HCC-induced rats with a combination of sorafenib and quercetin was more effective in restoring inflammation markers, the lipid profile and liver enzymes than single treatments. Furthermore, the combined treatment was more effective in inhibiting cancer progression and restoring key gene expression levels and the liver structural integrity. The sorafenib/quercetin combination is therefore a promising anti-cancer strategy to improve treatment outcomes.

## Figures and Tables

**Table 1:** Primer sequences used for reverse transcription polymerase chain reaction (RT-PCR).

Gene	Forward Primer (5′ to 3′)	Reverse Primer (5′ to 3′)	Ref.
***TNF-a***	5′CCCTGGTACTAACTCCCAGAAA-3′	5′TGTATGAGAGGGACGGAACC-3′	[31]
***VEGF***	5′ACAGAAGGGGAGCAGAAAGCCCAT-3′	5′CGCTCTGACCAAGGCTCACAGT-3′	[32]
***p53***	5′CCTATCCGGTCAGTTGTTGGA-3′	5′TTGCAGAGTGGAGGAAATGG-3′	[33]
***NFκB***	5′GCAAACCTGGGAATACTTCATGTGACTAAG-3′	5′ATAGGCAAGGTCAGAATGCACCAGAAGTCC-3′	[34]
***GAPDH***	5′CAACTCCCTCAAGATTGTCAGCAA-3′	5′GGCATGGACTGTGGTCATGA-3′	[35]

**Table 2:** Initial and end changes in body weight, liver weight and liver index in the study groups following HCC treatment (from week 7 to week 13) by sorafenib, quercetin or both molecules. Groups 1 and 2 are non-induced and HCC-induced rats, respectively. Groups 3, 4 and 5 correspond to HCC-induced rats treated with quercetin, sorafenib and sorafenib/quercetin, respectively.

Data	Group 1	Group 2	Group 3	Group 4	Group 5
**Initial body weight (grams)**	278.16 ± 29.56	247.19 ± 17.40 a	277.77 ± 29.24 b	263.74 ± 19.60	251.63 ± 15.73 a
**Final body weights (grams)**	319.58 ± 4.04	269.90 ± 33.36 a	304.84 ± 40.84 b	316.55 ± 26.53 b	290.09 ± 28.73
**Ratio body weight increase (%)**	14.89%	9.19%	9.75%	20.02%	15.28%
**Liver weights (grams)**	8.78 ± 1.31	7.98 ± 1.37	8.81 ± 1.39	8.50 ± 1.11	8.22 ± 0.62
**Liver index (%)**	2.75 ± 0.13	2.95 ± 0.29	2.89 ± 0.16	2.68 ± 0.22 b	2.84 ± 0.15

Data were expressed as mean +/− standard deviation. Significance was made using one-way ANOVA test followed by least significant test. a: significance versus group 1. b: significance versus group 2. Liver index = liver weight divided by final body weight × 100. Significance at *p* < 0.050. Ratio of total body weight changes (%) = body weight at 13th week—body weight at 7th week/body weight at 7th week × 100.

## Data Availability

Not applicable.

## Sample Availability

Samples of the compounds are not available from the authors.
